# Reconstructing Viral Genomes from the Environment Using Fosmid Clones: The Case of Haloviruses

**DOI:** 10.1371/journal.pone.0033802

**Published:** 2012-03-30

**Authors:** Inmaculada Garcia-Heredia, Ana-Belen Martin-Cuadrado, Francisco J. M. Mojica, Fernando Santos, Alex Mira, Josefa Antón, Francisco Rodriguez-Valera

**Affiliations:** 1 Evolutionary Genomics Group, División de Microbiología, Universidad Miguel Hernández, Alicante, Spain; 2 Departamento de Fisiología, Genética y Microbiología, Universidad de Alicante, Alicante, Spain; Auburn University, United States of America

## Abstract

**Background:**

Metaviriomes, the viral genomes present in an environment, have been studied by direct sequencing of the viral DNA or by cloning in small insert libraries. The short reads generated by both approaches make it very difficult to assemble and annotate such flexible genomic entities. Many environmental viruses belong to unknown groups or prey on uncultured and little known cellular lineages, and hence might not be present in databases.

**Methodology and Principal Findings:**

Here we have used a different approach, the cloning of viral DNA into fosmids before sequencing, to obtain natural contigs that are close to the size of a viral genome. We have studied a relatively low diversity extreme environment: saturated NaCl brines, which simplifies the analysis and interpretation of the data. Forty-two different viral genomes were retrieved, and some of these were almost complete, and could be tentatively identified as head-tail phages (*Caudovirales*).

**Conclusions and Significance:**

We found a cluster of phage genomes that most likely infect *Haloquadratum walsbyi*, the square archaeon and major component of the community in these hypersaline habitats. The identity of the prey could be confirmed by the presence of CRISPR spacer sequences shared by the virus and one of the available strain genomes. Other viral clusters detected appeared to prey on the Nanohaloarchaea and on the bacterium *Salinibacter ruber*, covering most of the diversity of microbes found in this type of environment. This approach appears then as a viable alternative to describe metaviriomes in a much more detailed and reliable way than by the more common approaches based on direct sequencing. An example of transfer of a CRISPR cluster including repeats and spacers was accidentally found supporting the dynamic nature and frequent transfer of this peculiar prokaryotic mechanism of cell protection.

## Introduction

Viruses are a part of the genetic baggage of prokaryotic species and, therefore, collecting genomes of viruses that infect a certain prokaryotic species is of paramount importance in understanding the genomic diversity of the host [2], [3], [4]. However, the problem of characterizing phages of cells with poor culturability is a major obstacle to properly describing the genomic diversity of these prokaryotic species. Metagenomics provide a way to bypass the difficulty of obtaining genomic information about microbes that are hard to retrieve in pure culture, and sequencing the metaviriome should help in complementing the genomic information provided by the metagenome [5], [6], [7], [8], [9]. There are large datasets of metaviriomes [4], [10], but they are mostly short reads that often cannot be assembled and are very difficult to study given the enormous diversity of the gene complement of viruses and the problems inherent in the annotation of viral ORFs. In addition, with a few exceptions, viral metagenomes are often obtained after amplification of environmental viral DNA using mostly two methods (linker amplified shotgun libraries or multiple displacement amplification) that have been shown to introduce different biases in the recovery of viral diversity [11]. One way to improve the descriptive power of metaviriome sequencing is by cloning the purified viral DNA in fosmid vectors. They pack insert sizes that are close to average sizes of viruses infecting prokaryotes i.e. *ca*. 40 kb and offer natural contigs much easier to interpret and analyze [12].

Here we have combined the direct cloning of environmental viral genomes with high throughput sequencing technologies to describe putative viruses in an extreme environment of restricted diversity: the NaCl saturated brines of the crystallizer CR30 of a solar (marine) saltern of Santa-Pola (SP) (Alicante, Spain). This same pond (CR30) has been studied for more than 30 years using several approaches including cultivation, PCR 16S rDNA amplification sequence analysis, fluorescent *in-situ* hybridization and metagenomics [13], [14], [15], [16], [17], [18], [19], [20], [21]. All these studies show that members of the square archaeon *H. walsbyi* always dominate the prokaryotic community in this pond, representing between 60 and 80% of the cells present there. Actually, the first strain of *H. walsbyi* (DSM 16790) to have its genome sequenced [22], and one of the first two isolates of this species [23], comes from CR30. Recently the metagenome of CR30 was described by direct DNA 454 pyrosequencing [21] from the same sample as the one used to construct the viral fosmid library described here. Analysis of the rDNA fragments rescued from the metagenomic reads confirmed the predominance of *H. walsbyi* (79%), followed by *S. ruber* (9%), *Halorubrum* sp. (4%) and other haloarchaea 5%. In this work, only 2% of the 16S rDNA fragments could not be classified to a high-level taxon. The presence of the recently described Nanohaloarchaea [24] was proven at lower salinities (19%) in the SP saltern [21], but not in the 37% brine of CR30.

Although NaCl saturated brines are one of the lowest diversity aquatic habitats on earth, it is well known that they harbor one of the highest number of virus-like-particles (VLPs) reported for planktonic systems, from 7.3×10^7^ [25] to 2×10^9^ VLP ml^−1^ in the crystallizer ponds [26] and the Dead sea [27] respectively. In salt lakes, haloviruses generally outnumber cells by 10 to 100-fold [28]. Since the crystallizer of the SP saltern is dominated by *Archaea* and more specifically by *H. walsbyi*, it is to be expected that most of the viruses found here should prey on this microbe. Unfortunately, the extremely slow and demanding conditions for growth of this microbe [23] have prevented thus far the isolation of its viruses. However, phages have been obtained as pure cultures from other haloarchaea for many years (*Halobacterium, Natrialba* sp., *Haloarcula* sp., *Haloferax* sp. and *Halorubrum* sp.), and some of them have been sequenced (see review [29]). Most are head-tail viruses with double stranded linear DNA genomes (such as in HF1 and HF2, phiH, phiCh1, psiM1 and BJ1) and many times a packaging model accounting for the partial circular permutation and terminal redundancy of the DNA has been suggested. However, other morphologies and DNA structures, e.g. spindle-shaped (His1 and His2), icosaedric (SH1) or pleomorphic (HHPV-1 and HRPV-1) or single stranded DNA as HRPV-1 have also been described.

The morphology of viral particles in saturated brines has also been studied directly by electron microscopy of crystallizer samples. It was shown that *Haloquadratum*-like cells (flat squares) are frequently infected by lemon-shaped viruses, normally with high burst sizes, up to more than 350 VLPs per infected cell [26]. Other works, like the metaviriome study in lake Retba (Senegal) showed that 46% of the virus- like particles were spindle-shaped, followed by spherical viruses (35%), filamentous viruses (13%) and no more than 1% had head-tailed shapes [30]. However, by sequencing 16S rDNA libraries from this lake, only 9% of the community was adscribed to *Haloquadratum*. Besides, viruses with other morphologies have also been detected infecting *Haloquadratum* [31], probably head-tail viruses (personal communication). Along the same lines, other hypersaline environments dominated by *Haloquadratum*-related lineages have a very low concentration of lemon-shaped viruses, as observed in a Tunisian coastal solar saltern (Boujelbene *et al.*, submitted) or some samples of CR30 [32]. At the moment of this work, the only spindle-shape viruses isolated in pure culture are the ones of the thermophilic archaeon *Sulfolobus* (*Fuselloviridiae* family).

Presently, only one putative halophage (the host remains unidentified) genome, EHP-1 [33] has been obtained by a culture-independent approach (again from CR30) We have cloned and sequenced 42 fosmids containing genomes from the dsDNA viral fraction collected from CR30, 14 of which could be clearly assigned to *H. walsbyi* viruses based on GC content, tetranucleotide frequency analysis and the presence of CRISPR protospacers [34]. In addition, we have identified two fosmids clusters that could correspond to viruses infecting organisms of the recently described *Nanohaloarchaea* cluster [21], [24].

## Results and Discussion

### General features and classification of the viral contigs

**Figure 1:**
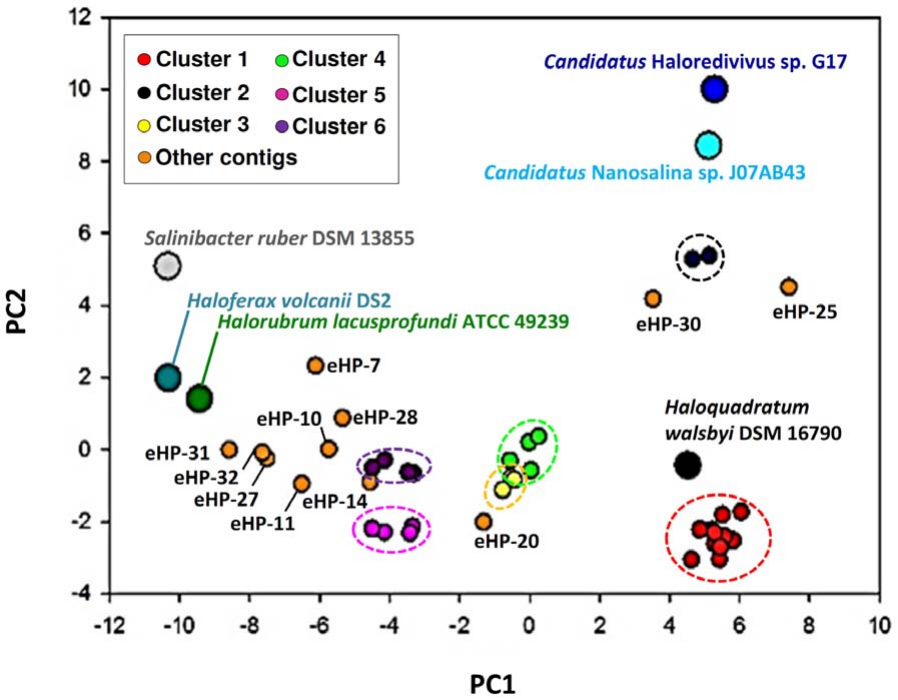
Principal component analysis of the codon usage of the assembled viral contigs and halophilic genomes. Complete genomes are shown as larger circles and the small dots correspond to the viral contigs. Different clusters are shown in different colours. In addition, clusters have been highlighted encircled by dashed-lines. Fosmids not part of any cluster are shown as “other contigs” in orange and labeled as in Table 1.

**Figure 2:**
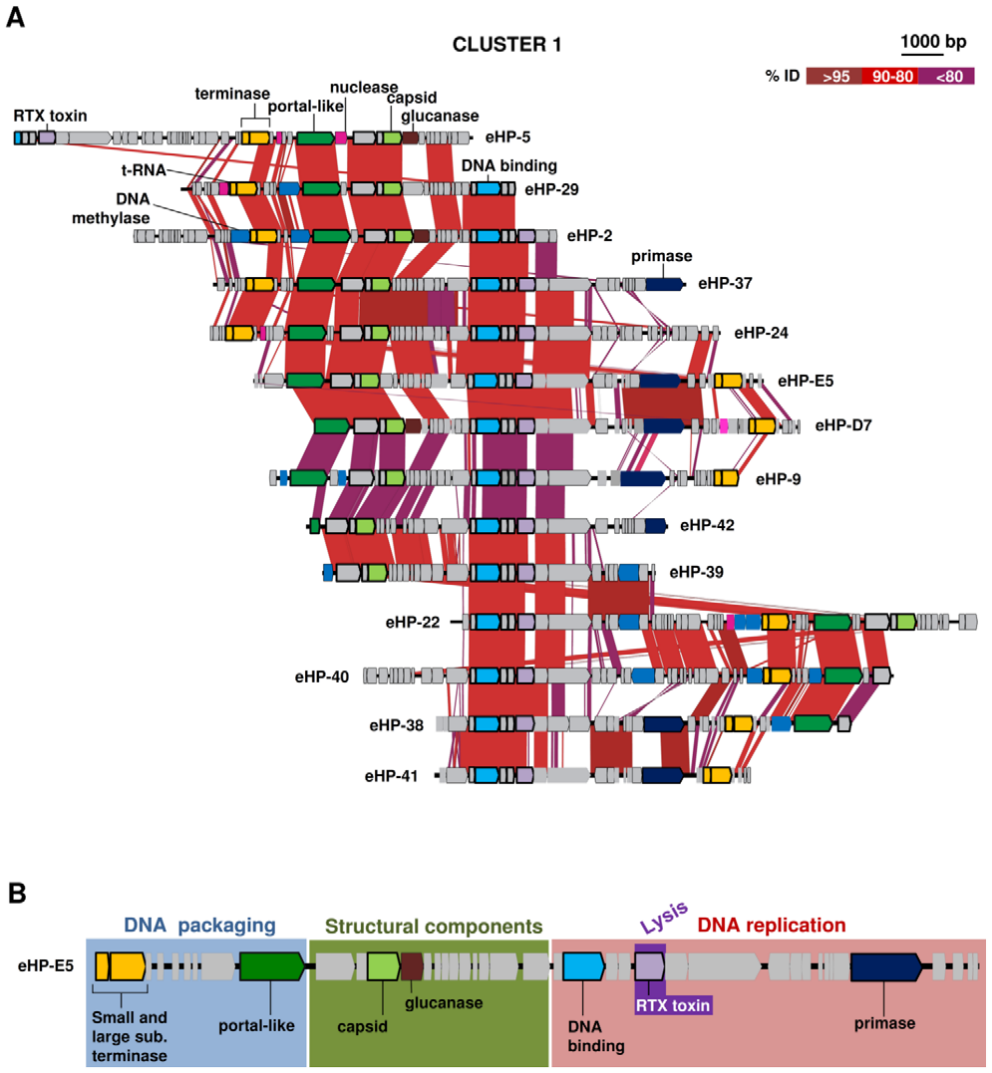
Comparative genomic organization of viral fosmids of cluster 1. Conserved genomic regions between fosmids are indicated by red shaded areas, red intensity being a function of sequence similarity by BLASTN. Specific ORFs mentioned in the text are labeled. Conserved ORFs are in bold. (B) Conserved modules in cluster 1 using eHP-E5 as a model. Gene colour-code is based on the functions assigned to the genes.

**Figure 3:**
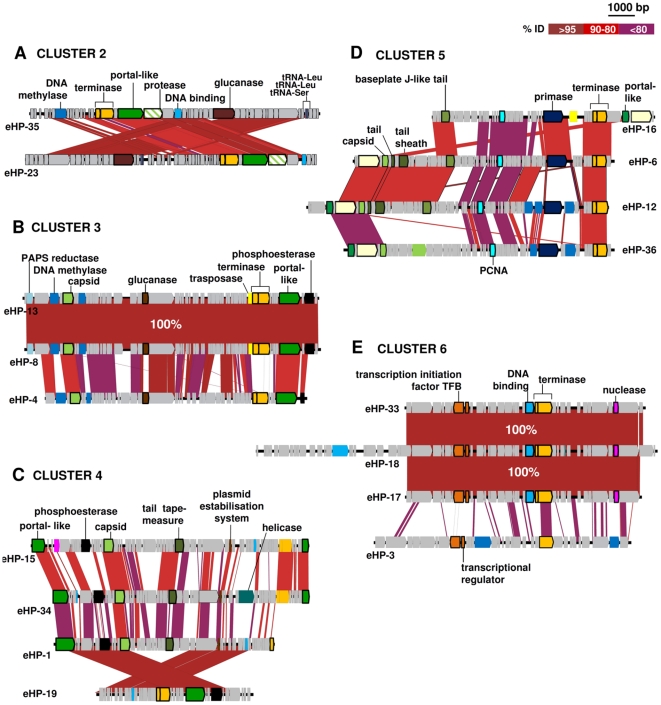
Comparative genomic organization of viral fosmids of cluster 2 (A), 3 (B), 4 (C), 5 (D) and 6 (E). Conserved genomic regions between fosmids are indicated by red shaded areas, red intensity being a function of sequence similarity by BLASTN. Specific ORFs mentioned in the text are labeled. Conserved ORFs are in bold. Colour code is same as Figure 1.

Viral DNA was extracted and fosmid libraries were constructed from two samples of the crystallizer pond CR-30 taken during summer and winter 2008. Two additional fosmids (eHP-D7 and eHP-E5) from a viral metagenomic library constructed previously (sample taken in spring 2007) from the same pond [20] were also sequenced. In total, 42 fosmids (ca. 1.2 Mb) representing partial to almost complete (see below) viral genomes were reconstructed. Table S1 supplies the annotation of all the ORFs detected. As shown in Table 1, the sizes of the viral genomic fragments sequenced ranged from 20.2 to 43.6 kb, which fall in the genome size range previously reported from viruses inhabiting CR-30 [20], [32], [35]. Therefore, we can safely assume that the contigs correspond to significant fractions of the genomes from virus particles present in the crystallizer water at the time of sampling. Also the fosmids covered the whole range of GC content (43.9% to 60.8%) characteristic of the cells known to be abundant in the saltern [19], [36] (Table 1). When we compared the viral DNA sequences, it was possible to classify 31 from the 42 contigs into 6 different clusters which shared more than 75% nucleotide identity over at least 3 kb. These six clusters were also supported by tetranucleotide frequency analysis and codon usage (Figures 1, 2, 3 and S1). We have used these parameters to tentatively assign hosts to the putative viruses. Although the similarity in the codon usage and tetranucleotide frequencies among viruses and their hosts has been very often observed [37], [38], and has been used to detect the putative hosts [20], [39], the method is not failsafe. There are cyanophages that carry their own tRNA genes and do not need to have the same codon usage of the host to infect a cell [40]. Also, even in the absence of tRNAs it is possible to find viruses with almost a 20% different GC content with their host e.g. His1 and His2 of *Haloarcula hispanica* [41]. However, In the case of cluster 1, host assignment by codon usage (Figure 1) and tetranucleotide sequence analysis was confirmed by the presence of a CRISPR protospacers in the contigs.

**Table 1:** Main features of the fosmids.

Cluster	Fosmid designation	Length	%GC	Putative host	Num. ORFs	Num. reads recruited SP metaviriome#	Num. reads recruited SD metaviriome$	Underrecruiting ORFs
	**eHP-2**	27204	43.90	*H. walsbyi*	42	45	2026	Glucanase/3HP
	**eHP-5**	29473	44.06	*H. walsbyi*	45	51	2099	Glucanase/2HP/2nuclease
	**eHP-9**	30090	45.79	*H. walsbyi*	39	14	624	Primase/3HP
	**eHP-22**	33770	43.79	*H. walsbyi*	51	52	2246	2Methyltransferase/2HP
	**eHP-24**	32681	44.19	*H. walsbyi*	51	50	2353	Nuclease/5HP
	**eHP-29**	21453	44.44	*H. walsbyi*	32	40	1272	Nuclease/tRNA/TerS/7HP
**1**	**eHP-37**	30300	44.81	*H. walsbyi*	40	47	2155	Primase/2HP
	eHP-38 (*)	26566	44.64	*H. walsbyi*	33	32	1408	2HP/primase/terminase
	eHP-39 (*)	21302	43.95	*H. walsbyi*	28	25	1696	2Methylase/3HP
	eHP-40 (*)	33481	44.07	*H. walsbyi*	55	45	1989	HP/methylase
	eHP-41 (*)	20197	44.84	*H. walsbyi*	29	26	1225	2HP/primase
	eHP-42 (*)	23125	44.85	*H. walsbyi*	31	23	1245	3HP/primase
	**eHP-D7 (+)**	31094	44.78	*H. walsbyi*	43	39	2033	Glucanase/5HP/Primase
	**eHP-E5 (+)**	32692	45.04	*H. walsbyi*	41	56	2265	2HP/Primase
**2**	**eHP-23**	31231	43.47	Nanohaloarchaea	47	0	0	-
	**eHP-35**	31263	43.68	Nanohaloarchaea	47	0	0	-
	**eHP-4**	30520	50.76	Nanohaloarchaea	49	1	0	-
**3**	**eHP-8**	34381	50.59	Nanohaloarchaea	57	1	0	-
	**eHP-13**	35126	50.60	Nanohaloarchaea	58	1	0	-
	**eHP-1**	29837	51.00	-	50	3	1	-
**4**	**eHP-15**	37310	51.57	-	68	2	1	-
	**eHP-19**	21190	51.67	-	39	3	0	-
	**eHP-34**	34179	52.29	-	55	1	1	-
	**eHP-6**	37376	56.76	-	58	19	443	-
**5**	**eHP-12**	27204	56.34	-	57	22	548	-
	**eHP-16**	29473	56.45	-	41	14	409	-
	**eHP-36**	30090	56.92	-	53	16	472	-
	**eHP-3**	33770	56.60	*S. ruber*	31	0	0	-
**6**	**eHP-17**	32681	57.02	*S. ruber*	34	0	1	-
	**eHP-18**	21453	57.62	*S. ruber*	59	0	1	-
	**eHP-33**	30300	57.19	*S. ruber*	35	0	1	-
**NC**	**eHP-7**	26566	58.56	*S. ruber*	42	0	2	-
	**eHP-10**	21302	59.99	*S. ruber*	44	0	56	-
	**eHP-11**	33481	58.49	*H. lacusprofundi*	35	0	0	-
	**eHP-14**	20197	57.82	*H. lacusprofundi*	57	0	0	-
	**eHP-20**	23125	52.09		58	1	203	-
	**eHP-25**	31094	44.28	Nanohaloarchaea	32	0	0	-
	**eHP-27**	32692	60.80	*S. ruber*	44	0	0	-
	**eHP-28**	31231	57.94	-	36	2	0	-
	**eHP-30**	31263	45.94	Nanohaloarchaea	60	2	0	-
	**eHP-31**	30520	62.36	-	48	0	1	-
	**eHP-32**	34381	60.36	*H. lacusprofundi*	56	0	29	-

(*) Samples recovered in January 2008. (+) Samples recovered in May 2007. Other samples recovered in June 2008.

NC: non-classified fosmids;

# SP metaviriome: Metaviriome from Santa Pola saltern CR30 [20];

$ SD metaviriome: Metaviriomes from San Diego high salinity [5]. HP: Hypothetical protein.

### Cluster 1: *H. walsbyi* phages

Fourteen sequences correspond unequivocally to phages of *H. walsbyi* since they contain proto-spacers of CRISPR repeats found in the genome of the isolate *H. walsbyi* C23 (see below). Besides, they also clustered with *H. walsbyi* by codon usage (Figure 1) and tetranucleotide frequencies (Figure S1). The viral genomes retrieved are represented in Figure 2A. The genomes are largely collinear indicating the genome is linear rather than circular. With the exceptions of *Halorubrum* phage HRPV-1 and *Haloarcula* phage HHPV-1 (both pleomorphic) and with circular genomes all haloarchaeal phage genomes known are linear [29]. Cluster 1 genomes share a remarkable synteny (Figure 2A) although gene order is sometimes rearranged in a way reminiscent of the circularly permuted gene order seen in some phages that replicate by the rolling circle mechanism [42]. Besides, they contain similar highly conserved genes (shown in bold in Figure 2), including a hypothetical protein with a DNA binding domain followed by two small hypothetical proteins exclusively found in this cluster, a gene annotated as a cytolytic toxin and another two hypothetical proteins (one of them with only the N-terminal domain partially conserved). Also very well conserved are the genes coding for protein distantly related to the portal protein of phage-Mu, and a capsid protein that are always present in the same order. The non-conserved regions of the fosmids in this cluster are often very rich in short hypothetical proteins. The order of genes involved in morphogenesis is a conserved feature in some viral groups such as tailed phages and prophages [42], [43]. It is noteworthy that the phages of cluster 1 have been retrieved from the three samples used in this work and are probably major components of the viriome like its putative prey (*H. walsbyi*) is of the prokaryotic community.

In 11 of these fosmids, there is a gene coding for a terminase large subunit (TerL) together with a small protein which has a DNA binding domain, is always upstream, and corresponds in all probability to the small subunit of this enzyme (see below). These two genes are particularly relevant since they are considered hallmarks of head-tail phages. Terminase enzymes are hetero-oligomers comprising a small and a large subunit and are components of the molecular motor that translocate genomic DNA into empty capsids during DNA packaging in the head-tail viruses, order *Caudovirales* (dsDNA viruses with a head-tail morphology) [44]. Actually, a search of terminase homologues has been used to identify tailed proviruses integrated in archaeal genomes [45]. It is remarkable that all the fosmids of cluster 1 (and all the other clusters described here, see below) possess both terminase subunits (eHP-42 and 39 did not have them but these genomes appeared to be incomplete). Although the small subunit (TerS) could not be identified by similarity, a small protein which contains two long helices (essential for the functionality of the small terminase subunit [46]) is always upstream of TerL. This gene is likely to be the TerS of cluster 1, albeit with little similarity to previously described TerS genes. On the other hand, since both large and small subunits are needed for a functional “normal” terminase, and we have found only homologues to the large subunit, we cannot rule out the possibility that a family of terminase-like proteins partially related to that of *Caudovirales* is present in other viral genomes with some relevant functions (as anticipated by its wide distribution in our metaviriome). However in view of all the other evidence this seems unlikely since many other lines of evidence point to the *Caudovirales* affiliation of cluster 1. The capsid gene found in cluster 1 shares a low but significant similarity with the GpE capsid from the *Natrialba* head-tail virus phiCh1 [47], [48]. Another finding that supports the idea that the viruses of cluster 1 are head-tail viruses is the large proteins (up to 800 aminoacids) found downstream of TerL (in green in Figure 2B). Similar proteins, with a domain of *ca.* 100 amino acids near the C-terminus that shows significant similarity to a morphogenesis protein (gpF) of phage Mu, are found downstream of TerL in methanococcal proviruses (psiM2, psiM100 and Msmi-Pro1) [49] and in *Natrialba* sp. virus phiCh1, all typical head tail viruses. These kind of proteins have been proposed to work as portal proteins, which are essential for tailed viruses development and infection [50]. Finally, the overall similarity found in the structure of viruses of cluster 1 with many *Caudovirales* viruses [50], where genes are clustered in three separate modules for DNA packaging, structural components and DNA replication module (Figure 2B), strongly suggests that these *H. walsbyi* phages belong to this type. Downstream from the capsid gene, three of the putative viral genomes had ORFs annotated as glucanases. These genes are found in plant and bacterial viruses and are involved in degrading the host cell wall either during virus release and/or is packaged in the virion particle and then degrade the polysaccharide envelope to allow virus entry into the cell [51], [52]. Although these genes appeared only in three of the contigs, similar genes were found in other clusters and in a previous metaviriome [20]. All known haloarchaea have glycoprotein S-layers, and often exopolysacharide containing, cell envelopes, thus the presence of glucanase genes in the viruses fits well with the cell biology of the putative host.

This was unexpected since tailed viruses, although present in a relatively high proportion in the crystallizer CR-30, are not the dominant morphotypes [26]. One possible explanation is that, although one of the protocols used here (for both 2008 samples) has often been applied to the retrieval of environmental virus particles in metaviriomic studies, it is based in the lambda bacteriophage CsCl purification, a head-tail virus. Therefore it is possible that the protocol is biased to retrieving these kinds of viruses. However, comparison of the sequences of cluster 1 against the metaviriome of CR30 [20] point out against the existence of this methodological bias. Part of that metaviriome was constructed using a different methodology without CsCl gradient purification (see Materials and Methods). From a total of 22 fosmids-ends, 14 (63.6%) have a significant similarity with some region of a cluster 1 fosmid, which means that these head-tail viruses are frequently retrieved even with other isolation protocols. In any case, recruitment studies leave little doubt about the high prevalence of cluster 1 viruses in the CR30 and other saltern brines (see below).

#### CRISPR related elements found in cluster 1

Most sequenced archaeal genomes contain at least one CRISPR/Cas system [53], [54]. These genetic landmarks are composed of one or more arrays of short (most in the range 23–38 bp) regularly spaced direct repeats called CRISPR (Clustered Regularly Interspaced Short Palindromic Repeats) and a variable set of *cas* (CRISPR associated) genes [55]. Repeats are separated by sequences (known as “spacers”) that derive from other sequences (i.e. “proto-spacers”) located outside CRISPR loci, notably in viruses and plasmids of the microbe carrying the spacer. Spacers are considered to be either copied or transferred into the CRISPR array from the foreign element during an unsuccessful attack [56], [57], [58], [59]. Furthermore, the possession of spacers homologous to invader DNA molecules protects the cell against further infection by the alien element [56], [60]. Thus, for a given isolate, the presence of a spacer homologous to a sequence in a mobile element, such as a virus, is a strong indication that the strain has been a host of the infectious element. So far, there are two cultured representatives of *H. walsbyi*: strains C23 and HBSQ001, isolated respectively from Australian salterns and from the crystallizer CR30 (Spain) [23], [34]. The analysis of their genomes revealed that C23 contains two CRISPR systems, belonging to subtypes I-D and I-B respectively according to the current classification [61], while HBSQ001 only contains remnants of the I-B system [34].

**Figure 4:**
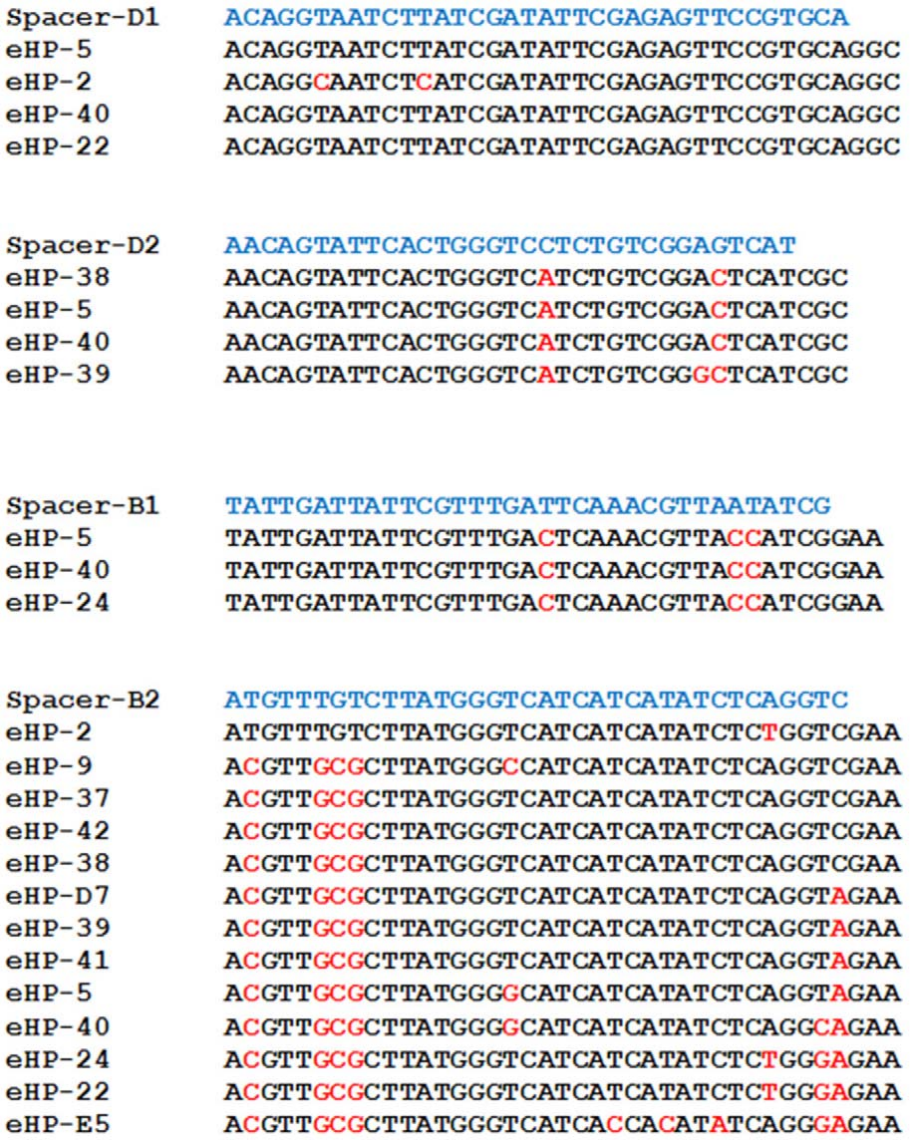
Alignments of CRISPR spacers of *H. walsbyi* C23 and homologous sequences (proto-spacers) found in our metaviriome. Putative PAM positions at the 3′ end of the proto-spacers are also included. Proto-spacers are identified by the name of the carrier fosmid and spacers by the letter of the corresponding CRISPR/Cas subtype. All proto-spacers have the PAM inferred from the analysis shown in Figure S2. PAMs (i.e. NGG or GAA for I-D and I-B systems respectively).

The 85 CRISPR spacers present in *H. walsbyi* C23 were compared against our fosmid sequences as described in Material and Methods. These BLASTN searches revealed sequences, located in fosmids eHP-2, 5, 22, 24, 38, 39 and 40 with identities over 90% to 4 *H. walsbyi* spacers (Figure 4). This is the identity threshold established to consider a sequence as a proto-spacer [58], [62], [63], [64]. Additional fosmids carrying sequences with lower identity to spacers (75–89%) were also detected (eHP-9, eHP-37, eHP-41, eHP-42, eHP-D7 and eHP-E5). All the fosmids in our metaviriome harboring putative *H. walsbyi* proto-spacers are included in cluster 1, which reinforces the hypothesis that this cluster contains viruses infecting *H. walsbyi* assemblages. This is remarkable considering that strain C23 was isolated from salterns located more than 16,000 km away from CR-30.

For a CRISPR system to confer immunity, the occurrence of a short motif (named PAM for Proto-spacer Adjacent Motif; [62] next to a particular end of the proto-spacer is required [65], [66]. The PAM sequence depends on the CRISPR/Cas variant [62], [67], [68], [69]. Thus, the presence of the corresponding PAM adjoining a sequence homologous to a given putative proto-spacer [62] supports (i) that the sequence is indeed a proto-spacer and (ii) the element carrying the sequence is infecting the host harboring the spacer. In order to identify PAM motifs for each of the two CRISPR/Cas systems of *H. walsbyi*, we aligned regions containing those proto-spacers with over 90% identity to spacers previously identified by Dyall-Smith and coworkers (2011). The conservation of the di-nucleotide GC was observed one position after the proto-spacers of the I-D system, and the tri-nucleotide GAA was found adjacent to the proto-spacers of the I-B system (Figure S2). In both cases, the orientation of the PAM with respect to the spacers in the CRISPR arrays (proximal to the leader) concurred with that of the motifs previously defined for type I CRISPR/Cas systems [61], [62]. As expected, the proto-spacers of the 4 spacers mentioned above had the corresponding PAM in the correct position (Figure 4). These data strongly support the hypothesis that the viruses carrying these spacers infect *H. walsbyi*, and stand for a CRISPR-mediated interference of strain C23 against them.

### Cluster 2

Cluster 2 includes two low GC fosmids, eHP-35 and eHP23 (Figure 3A), whose genomes might be circularly permuted as the order of the genes was completely rearranged by translocation in the two fosmid sequences. According to the oligonucleotide composition (Figure S1), they could be associated to the only other low GC archaeal group that has been detected in saturated brines, the Nanohaloarchaea [21], [24]. This association was less clear by codon usage analysis (Figure 1). However, this new archaeal group is only represented by three genome fragments, none of which come from a solar saltern crystallizer. Recruitment analysis with the available genomes (that come from hypersaline lake Tyrrell (Australia) [24] and an intermediate salinity pond in the Santa Pola saltern) against the CR30 metagenome [21] indicated that distantly related Nanohaloarchaeal cells are found in significant numbers in CR30 (identity *ca.* 80% to any of the available genomes). Therefore it is possible that these two phages infect new groups of Nanohaloarchaea not yet revealed. This could also be the case for the eHP-30 and eHP-25, that although do not form a cluster, are relatively close to the Nanohaloarchaea by tetranucleotide frequencies and codon usage.

The genes found in the two fosmids in Cluster 2 are very similar (average 94.59%) and they contain the hallmark terminase gene also preceded by the small gene that codes for the putative small subunit like those of cluster 1. Therefore, they also could be members of the *Caudovirales,* as they share a similar order of the genes (terminases- and portal protein [50]. Another interesting gene found in this cluster is the prohead protease that is in the same order as observed in many *Caudovirales* relative to the terminases and the portal protein. Again, here glucanase genes followed the portal protein, what might be taken as an indication that the Nanohaloarchaea also have a polysaccharide containing cell wall. Very little is known about this new group of halophiles.

### Clusters 3–6 and unclassified fosmids

#### Low GC fosmids

By tetranucleotide analysis there are other two low-GC clusters, 3 and 4, with no host assignation (Figure S1) and together with eHP-20 (Figure S4), they form a tight group by codon usage (Figure 1). Cluster 3 is formed by three fosmids (Figure 3B), two of them eHP-13 and eHP-8 are completely synthenic and share 100% identity. In the third one, eHP-4, the conservation is reduced to some genes. However, the capsids and also the putative portal protein are well conserved as found in cluster 1. At the 5′ end of eHP-13 and 8, there is a phosphoadenosine phosphosulphate (PAPS) reductase similar (51%) to the one found in the *Rhodococcus* phage RequiPine5 [70], also a member of the family *Caudovirales*. One of the genes that could help to affiliate this group of sequences is the gene with a calcineurin-like phospho-esterase domain found at the 3′ terminus. As suggested before [71], this domain is very well conserved in the small subunit of archaeal DNA polymerase II. The domains of the genes found in the fosmids of cluster 3 have a similarity of 37 and 40% to the DNA polymerase II of *Nanoarchaea equitans* Kin4-M and the one of *Candidatus* Nanosalina respectively. This suggests these phages also infect the Nanohaloarchaea.

Cluster 4 (Figure 3C), also contains the terminase genes (except in eHP-15 that is probably truncated and in eHP-1 where only the small subunit is conserved). On the other hand, the F-like protein (portal like) at the 5′ terminus is conserved in all of them. The flip observed in eHP-19 and the conserved regions between the fosmids suggest that their genomes could also be circularly permutted. All the contigs in this cluster have, like in cluster 3, the gene with a calcineurin-like phospho-esterase domain. However, this gene belongs to a non-conserved metallophosphatase not found in DNA polymerases and with no similarity to nanoarchaeal genes. Also, it is worth mentioning the presence of a gene coding for a plasmid stabilization system of similar length in all of the genes in the contigs of cluster 4 as well as in the non-clustering fosmid eHP-10 (Table 1). Members of this family are described as “plasmid stabilization protein” although the exact molecular function of these proteins remains largely unknown (Boujelbene *et al.*, submitted). Homologues have been found in bacterial and archaeal genomes as well as in 6 bacteriophages (*Burkholderia* phages phi644-2, phiE125, and phage phi1026b, *Mycobacterium* phage Fruitloop, *Mannheimia* phages phiMhaA1-PHL101 and phiMhaA1-BAA410), all of them of the *Caudovirales* family. eHP-34 has a helicase, which are proteins very well conserved in archaea and eukaryotes, but also are present in other head tail halophages such as BJ1 that infects *Halorubrum* [72]. All these data suggest that, again, phages from cluster 4 are head-tail viruses.

#### High GC fosmids

By tetranucleotide analysis (Figure S1), cluster 5 could not be assigned to any host, but there is protein near the 5′ terminus in eHP-12 that has a 67% of similarity to the protein coded by an ORF of the 47 Kb plasmid pL47 (HQ4002A) of *H. walsbyi* DSM 16790. However, no other similarity was found along the plasmid. As it was found in cluster 1, the fosmids of cluster 5 also have the primase subunits and as suggested by the order rearrangement of the ORFs, they are also viruses with circular or circularly permuted genomes (three of them eHP-16, eHP-6 and eHP36 have similar structure) (Figure 3D). In addition to the conserved terminases and the presence of capsids, a tail protein with a domain only found in *Caudovirales* (56% similarity to the *Streptomyes* phage mu1/6) was found here. A tail sheath protein 42% similar to the *Halorubrum* phage HF2 and a base plate J-like protein leave little doubt about the head tail nature of these viruses. The presence of these proteins suggests that cluster 5 could correspond to phages with a more complex structure. In complex phages, like T4, tails are surrounded by a sheath that contracts during infection, and at the end of the tail they have a base plate and one or more tail fibers attached to it. The base plate and tail fibers are involved in the binding of the phage to the bacterial cell. Downstream of these tail proteins, a phage late control D protein, which is needed for the lysis of the cell, was detected. These data point out that these viruses could be lytic phages and might explain the high recruitment observed in the metagenomes (Table 1) (see below), particularly considering that if the host is a high GC microbe, it cannot be very abundant in CR30. In addition to the “normal” viral proteins, we also found some unexpected gene products such as the proliferating cell nuclear antigen (PCNA) homologues found in all the viruses of this cluster (Figure 3D) (plus eHP-11 and eHP-14, outside this cluster). This protein is a non-histone acidic nuclear protein that plays a key role in the control of eukaryotic DNA replication. Homologues of PCNA have also been identified in the Archaea as well as in dsDNA viruses infecting different phototrophic and heterotrophic protists and in virus phiCh1 infecting the haloalkalyphilic archaeon *Natrialba magadii* [48].

#### CRISPR related elements found in cluster 5

Most CRISPR have been found in chromosomal regions unrelated to mobile elements [53]. Only occasionally, CRISPR/Cas components are located in plasmids and prophage related sequences [73], which could mediate their spread by lateral gene transfer (LGT). Indeed, LGT of CRISPRs has been observed between distant taxonomic groups [74], [75], [76]. The search of CRISPRs in our metaviriome revealed an array with five repeats with the corresponding four spacer sequences (Figure S3). This repeat-spacer cassette is found also in fosmid eHP-16, which is included in cluster 5. In an attempt to identify the origin of these CRISPRs, BLASTN analyses were carried out against the nr/nt database at the NCBI web site (http://www.ncbi.nlm.nih.gov/BLAST/). While spacers did not have any significant match in the database, repeats were related to those of haloarchaeal genomes (Figure S2). The presence of CRISPR in the viral genome can be explained through an LTG event from the host to the virus in a previous infection event. Many viruses acquire genes from their host along the infection cycle [77], a phenomenon that in many instances has been proven to confer advantages to the virus [78]. In this case, although we are not able to envisage any putative advantage for eHP-16, the presence of the CRISPR system can be used to make an assignment of its putative host. Most likely, ancestors of eHP-6 have infected high GC haloarchaea such *Haloferax* or *Natronomas* species, both haloarchaea with GC ranging within the values found for cluster 5. So far, the presence of CRISPR systems in free viruses had been detected in the human gut virome [79] and was also reported in a potential prophage found in the genome of *Clostridium difficile* [73]. This third report underscores the relevance of viruses as gene transfer agents for CRISPR cassettes.

Fosmids of cluster 6 (Figure 3E) together with other three unrelated fosmids (eHP-7, 10 and 27, (Figure S4)) cluster with *S. ruber* DSM 13855 by tetranucleotide analysis (Figure S1) but this affiliation could not be confirmed by the codon usage that was different (Figure 1). Three of the four fosmids of this cluster (eHp-33, 18 and 17) are nearly identical (100% identity in 20 kb). The fourth, eHP-3, is distantly related and the conserved region is reduced to the terminases and a few domains of hypothetical proteins. Most of the predicted proteins of this cluster contigs lacked homologues in the public databases. It is remarkable the degree of conservation of the three nearly identical contigs found, suggesting that this single clonal virion is very abundant or has been recently been released *en masse* from a population of prey cells. If they really prey on *S. ruber,* an organisms that appears to be always a minor component of the population, they might be under a lower pressure to change, since probably the host cells are also less diverse than other more abundant dwellers such as *H. walsbyi* [1].

Other not classified fosmids of high GC are eHP-11, 32 and 14. All are grouped together by tetranucleotide frequency with *Halorubrum lacusprofundi* ATCC 49239 but, again, the codon usage analysis shows them to be distinct. Only two of the 42 analyzed fosmids, eHP-31 and eHP-32, outside the 6 main clusters described above, harbor genes coding for integrases. Thus, according to these results and if we assume that most of the fosmids represent almost complete viruses, only a small proportion of the viruses present in the crystallizer at the time of sampling have the potential to undergo a lysogenic cycle. This is in agreement with a previous study of the viral metagenome of the same crystallizer in which also a small number of genes coding for integrases were found [20]. This, however, does not rule out the possibility that the viruses studied here carry out chronic infections in which viruses extrude continuously from the cell without causing lysis. In fact, chronic infections [29] have been proposed to be the most prevalent type of infection for archeoviruses [80], that most likely dominated viral communities in hypersaline environments, although so far there is no direct evidence of such prevalence in natural environments.

### Comparison of the clusters to each other and to hypersaline metagenomes

**Figure 5:**
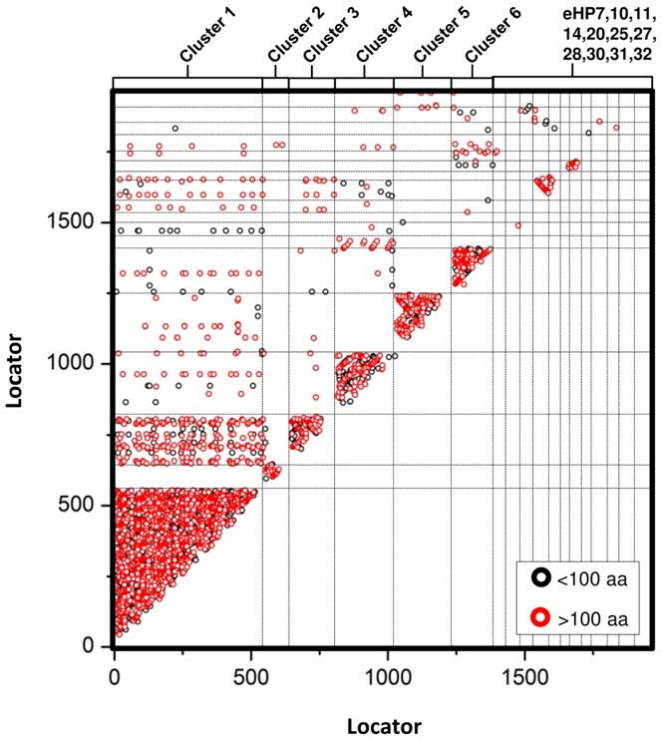
Self-against-self. BLASTP searches within all the contigs. ORFs are referenced by a numeric label ranging from 1 to 1914 (“locator” in the axis) and ordered by clusters (1–6). Black dots represent BLASTP matches showing an alignment length below 100 bp, while red dots show alignments equal to or greater than 100 bp. Vertical lines indicate the clusters (1–6).

**Figure 6:**
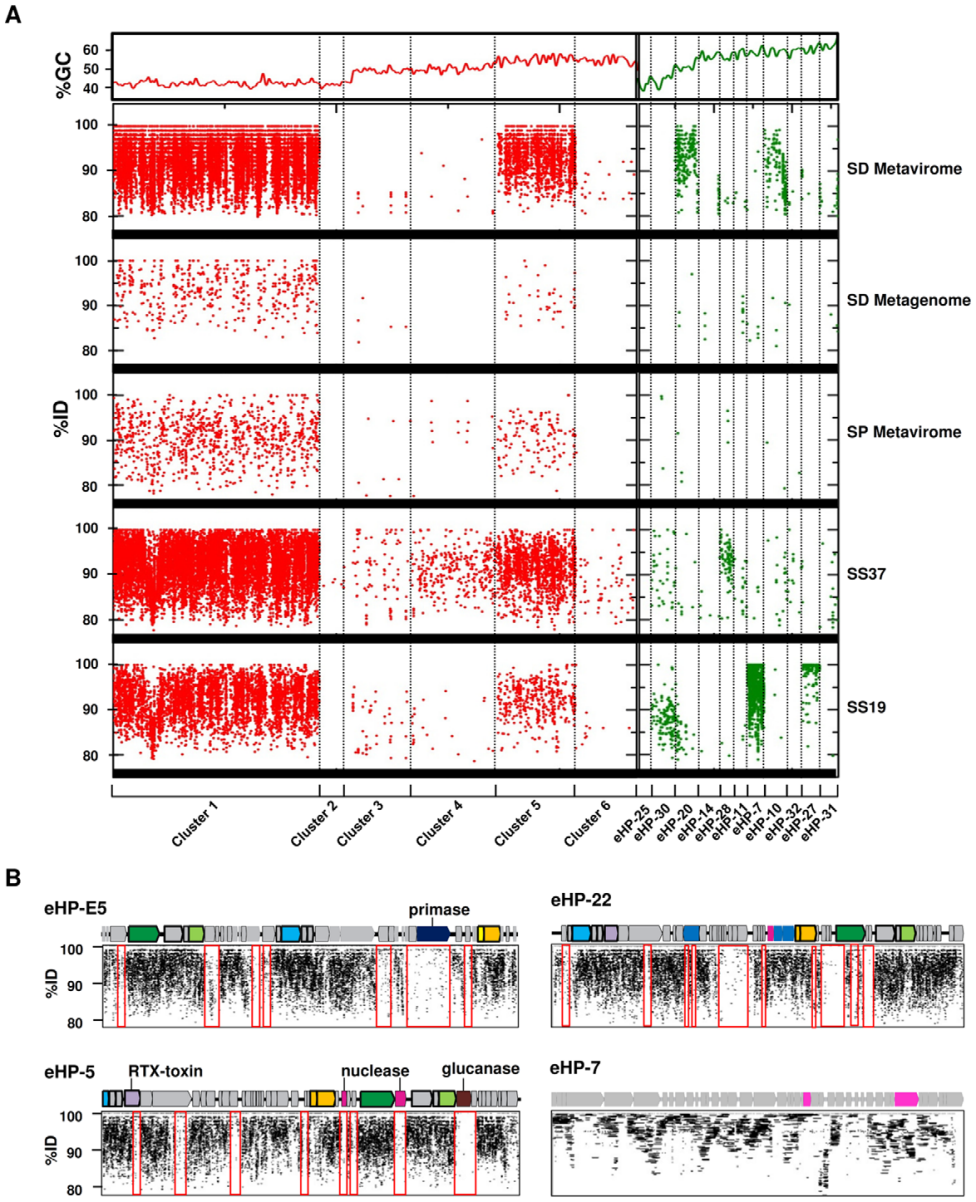
Recruitments of environmental datasets by the viral fosmids. (A) Recruitment of the environmental reads of Santa Pola (SS19, SS37 [21] and SP metaviriome [20]) and San Diego (SD metagenome and metaviriome [5]) saltern by the viral fosmids. An artificial concatenate of ORFs were constructed and BLASTN was used to make the comparison. The order follows increasing values of the GC content (upper panel). Vertical lines separate the clusters 1–6 (in red) and the not classified ones (in green). (B) Recruitments of San Diego metaviriome by the fosmids eHP-E5, eHP-5, eHP-22 and eHP-7. Underrecruiting islands are indicated by red rectangles and the function of the genes is indicated when known. Colour code as in Figure 1.

All the viral sequences were compared to each other (Figure 5) and to previously published viral and cellular metagenomes from hypersaline environments (Figure 6A). Self-to-self analysis returned a total of 1162 hypothetical proteins that were conserved in different viral genomes and should thus be considered as conserved hypothetical proteins [20]. In this way, the amount of HP provided by the automatic annotation was reduced in 65%. Approximately 75% of these new HPs turned out to be specific of a given cluster (346 of cluster 1, 77 of cluster 2, 109 of cluster 3, 131 of cluster 4, 126 of cluster 5 and 86 of cluster 6). It is worthy to note the high number of cluster specific proteins among the complete set of conserved HP in fosmids. Some clusters shared very few ORFs with the rest, such as cluster 2 which did not have hits with any other cluster.

The fosmids were also compared with previously published cellular and viral metagenomes from two multipond solar salterns located in San Diego, California [5] and Santa Pola, Spain [20], [21] (Figure 6A). The two samples from Santa Pola saltern corresponded to two ponds of 19% (SS19) and 37% salinity (CR30, identified as SS37 in [21]). The 37% CR30 salinity sample was the same as the June 2008 sample used in this work, as described in the Materials and Methods section. At first glance, it is obvious from Figure 6A that there are discontinuities in contig recruitment patterns between the different clusters partially associated to their GC content (upper panel in the figure). Low GC contigs recruited much more than high GC ones what is concordant with the dominant GC content of the dominant members of the population [19], [36]. The cluster that displays a higher number of hits in the analyzed metagenomes is cluster 1 (Table 1), in good agreement with the high abundance of *H. walsbyi* in these systems. However, fosmid eHP-9 recruited significantly fewer hits indicating either uneven densities or that the differences in the genome (Figure 2A) prevent cross-recruitment with other viriotypes. Fosmid eHP-7 that might prey on *S. ruber*, shows a remarkably high recruitment from the metagenome SS19, and only a few hits from SS37 (Figure 6A, 6B and Table 1). The microbial community of these two ponds has been analyzed by an in-depth metagenomic study [21] and the number of environmental hits to *S. ruber* type strain genome with SS19 (19% salinity) was two-fold the hits to the CR30 (SS37) metagenome. All these data suggest that eHP-7 may be infecting bacteria closely related to *S. ruber*. However, there are other *Bacteroidetes* in SS19 related to *Salinibacter* that could be acting as host for eHP-7. This could be also the case of contigs eHP-10 and eHP-27 that, to a different extent, follow the patterns described for eHP-7.

As the recruitment was abundant in the SD metaviriomes and the CR30 cell metagenome for the fosmids of cluster 1, individual virus genome recruitments assays could be carried out as the examples shown for eHP5, eHP-E5 and eHP22 in Figure 6B. The lack of even recruitment by the phage genomes, i.e. some genes recruit much more and at much higher similarity than others, is immediately obvious. This is reminiscent of the metagenomic islands described for genomes of bacterial or archaeal strains [19], [81], [82] that has been shown to be a widespread phenomenon at least in aquatic habitats [1], [83]. In cellular genomes the islands often code for phage receptors exposed on the cell surface and have been postulated to provide diversity of targets to distribute the load of phage predations among the different clonal lineages in the population. This model has been termed constant-diversity and was recently supported in a *Prochlorococcus* model system [84]. This model would also predict that a similar diversity would be found in the phages at the level of adapting to the different clonal cellular lineages. Along these lines we have found some under-recruiting genes that indicate a clear correlation with host recognition. The glucanase of cluster 1 was under-recruiting in all the genomes in which it was found. This would be a gene for which a high level of diversity would be essential to recognize the diversity of cell envelope polysaccharides of the putative hosts [81]. The difficulty of annotating many of the phage ORFs found here precludes more refined analysis, i.e. many of the under-recruiting ORFs are only HPs. However, a few other examples were detected. The second part of the gene annotated as RTX toxin might be involved in cell lysis and slight differences in the intracellular environment of the host might require different versions. The same can be said about nucleases, primases and methylases all of which under-recruited totally or partially.

### Conclusions

Using a combination of cloning in fosmids and high throughput sequencing we have obtained the sequence of 42 almost complete viral genomes directly retrieved from the metaviriome. Previous metaviriomes, even from low diversity environments did not allow assembly of near complete genomes. Therefore, although this approach might be biased by the limitations of fosmid cloning, it appears as the most productive in terms of information about the viral population in an environment. We could assign many of the viruses to a putative host and also to infer the type of virus.

We have been able to describe a new group of phages that prey on the square archaeon *H. walsbyi*, the predominant microbe in saturated NaCl brines. The presence of CRISPR protospacers in some of the phages in this cluster prove this association and could also help identify the natural host of other environmentally extracted viral entities. To the best of our knowledge, this is the first metagenomic study analyzing the diversity of viruses infecting a specific microbe using a culture-independent approach. Along the same lines, we have been able to detect groups that probably prey on *S. ruber* and the newly described, and as yet uncultured, Nanohaloarchaea, covering thus most of the abundant cellular types that are found in this environment. Some CIRSPR sequences described in *Haloferax* and *Natronomonas* genomes were also found in the genomes of viruses of cluster 5 what could be taken as indication that these viruses prey on the high GC haloarchaea. Additionally this finding indicates a role of viruses as carriers of CRISPR elements (both the tandem repeat and the spacers) by lateral gene transfer.

In spite of the predominance of archaea in the cellular community of the saturated brines, all the viral genomes described here have tell-tale features that are typical of the head-tail phages *Caudovirales,* the most common type of bacterial phages. More characteristic archaeal phages, such as the spindle shaped *Fuselloviridae,* were not retrieved by this approach, a phenomenon previously observed in other halophilic metaviriomes [20], [30].

The variability of the recruitment efficiency of the reconstructed phage genomes from metaviriomes of short reads allows detection of genome regions that are highly variable. We have found high variability of genes such as glucanases that are clearly associated to variation in the exposed cell structures of the host. This indicates a high diversity of viral clones that are different at the level of host recognition features [1]. The high recruitment of viral genomes from cellular metagenomes indicates that a high number of viruses is contained inside the cells at the sampling time and that the viruses are undergoing a lytic or a chronic infection cycle rather than lysogenic what is in good agreement with the lack of integrase genes found in cluster 1.

## Materials and Methods

### Sampling and isolation of viral DNA

Water samples were filtered sequentially through 20, 5 and 0.22 µm (Millipore, Westborough, MA, USA) from the crystallizer CR30, Santa Pola, Spain (38°11′47.33‴N, 0°35′00.80″W) on May in 2007 and January and June 2008. All necessary permits were obtained for the described field studies. The salinity was measured with a hand-refractometer and was 32%, 32% and 37% respectively. Before viral DNA was extracted, a treatment with DNAse and RNAse was carried out in all the samples. For the first sample, the 0.22 microns filtrate was concentrated by tangential flow filtration (TFF) through a 100-kDa filter cassette (PTHK00005) with a Pellicon System (Millipore) followed by ultracentrifugation (288 000 g; 3 h at 10°C; Optima XL Series, Beckman Coulter with a SW41TI rotor). Viral DNA was extracted, checked for quality by pulsed field gel electrophoresis (PFGE), purified and cloned in fosmids as described before [33]. For the samples of 2008, the virus-containing filtrate was concentrated to a final volume of ∼200 ml using a 100-kDa TFF filter (Millipore, Westborough, MA, USA) and a cesium-chloride gradient was used for the isolation of the phage particles as described in [6]. The viral DNA was isolated by formamide lysis and cetyl-trimethylammonium bromide extraction [85].

### Construction of the viral fosmid library

For each sample, a fosmid metagenomic library was constructed using the CopyControl® Fosmid Library Production Kit (Epicentre) following the directions of the provider. 30 to 40 kb metagenomic DNA fragments were cloned in the pCC1Fos vector and replicated in *Escherichia coli* EPI300. A total of 23 fosmids were obtained for the sample of May 2007. In the case of the 2008 samples, a total of 65 clones were obtained for the sample of January and 1248 for the one of June 2008.

### Sequencing and assembly

Fosmids were selected randomly for complete sequencing, 65 from the winter 2008 library, 90 from the summer 2008 and 2 from May 2007. They were individually grown and induced to high number copy. The fosmid DNA was extracted using QIAprep Spin Miniprep kit (QIAGEN). DNA was checked for quality on a 1% agarose gel and measured using Quant-iT® PicoGreen ® dsDNA Reagent (Invitrogen). DNA was sequenced in a half run of the Roche 454 GS-FLX system (GATC, Konstanz, Germany), pooling 12–13 fosmids together and tagging each group individually using a multiplex identifier adaptor. Additionally, one Solexa lane was performed to increase the coverage and correct the 454 errors (Macrogen, Corea). Two different programs were used in the assembly, Geneious Pro 5.0.1 (with default parameters (http://www.geneious.com)) and MIRA [86]. Only contigs confirmed by both programs were considered. Thus, 42 fragments larger than 20 kb were finally obtained with a coverage range between 20–30×. The two of the clones from the sample of spring 2007 were completely sequenced in an independent “run” using the Roche 454 GS-FLX system. In this case, the coverage was of 25× for eHP-E5 and 28× for eHP-D7.

### Analysis of the sequences

GC content was calculated using the EMBOSS tool *geecee* [87]. Tetranucleotide frequency of the viral fosmids and the related sequences were done using the on-line tools in http://insilico.ehu.es and a dendrogram was constructed applying the UPGMA clustering of the Euclidean distance of the frequencies. Codon usage of the viral fosmids and the putative hosts was calculated with the EMBOSS tool *cusp* [87] and a principal component analysis (PCA) was carried out using R 2.13.0. Gene prediction on the assembled contigs was done using MGA [88]. The predicted protein sequences obtained were compared using BLASTP to the NCBI nr protein database (e-value 1e-5) (http://www.ncbi.nlm.nih.gov/BLAST/). ORFs smaller than 100 bp and without significant homology to other proteins were rejected. To confirm the presence of domains in the predicted proteins the hmmpfam program of the HMMER package [89] (e-value 1e-3) was used and the hmm models for the protein domains were obtained from the Pfam database (http://pfam.sanger.ac.uk). Also, different searches were done with InterProScan (http://www.ebi.ac.uk/Tools/InterProScan/) and the Conserved Domain Database (http://www.ncbi.nlm.nih.gov/Structure/cdd/wrpsb.cgi). Self-against-self comparisons were done matching all the ORFs against themselves using BLASTP. Significant matches were considered as those having a minimum identity of 60% and a minimum alignment length of 50 positions (e-value 1e-3). ACT Artemis.v12 [90] and perl-software developed in our laboratory was used to compare the viral sequences among them. Also for comparative analyses, reciprocal BLASTN and TBLASTXs searches among the different fosmids were carried out, leading to the identification of regions of similarity. CRISPR arrays were identified using the CRISPR-finder program available at the web site http://crispr.u-psud.fr/ [53], and putative proto-spacers by BLASTN searches with spacers as query. For the identification of the proto-spacer adjacent motifs (PAMs) of the two CRISPR/Cas systems of *H. walsbyi,* regions containing proto-spacers with over 90% identity to spacers of strains HBSQ001 and C23^T^ were obtained from the nr database and the strands complementary to their corresponding crRNA were aligned with the WebLogo application (http://weblogo.berkeley.edu/logo.cgi), using equivalent ends (with respect to the CRISPR sequence) of the spacers as a reference.

### Recruitments of environmental collections

Different recruitment plots against available halophilic metagenomes and metaviriomes were done using BLASTN [91] with a cut-off of 75% of identity in 50% of the length of the environmental read. For recruitment analysis we used metaviriomes and metagenomes recovered from the same crystallizer CR30 (SS37) from which the viral DNA was isolated and another pond from the same saltern and lower salinity (SS19), [20], [21]. Besides, we also used the metaviriomes from the salterns of San Diego (California, USA) [5].

Sequence data have been deposited in the Genbank under the BioProject ID: PRJNA82917.

## Supporting Information

Figure S1 **Dendogram showing the distribution of viral sequences according to their tetranucleotide frequency.** Oligonucleotide analysis of the fosmids was done using the on-line tools in http://insilico.ehu.es and a dendrogram was constructed applying the UPGMA clustering of the Euclidean distance of the frequencies. In red, the prokaryote genomes and in bold, the viral fragments sequenced in this work.
(TIF)

Figure S2 **Identification of proto-spacer adjacent motifs (PAMs) of the two CRISPR/Cas systems of** ***H. walsbyi*** **.** Regions containing proto-spacers (positions ^−^33 to 0) with over 90% identity to subtype I-D (6 entries) or I-B (9 entries) spacers of strains HBSQ001 and C23^T^, were obtained from the nr database (http://www.ncbi.nlm.nih.gov/BLAST/). Proto-spacer sequences where aligned with the WebLogo application (http://weblogo.berkeley.edu/logo.cgi) using the CRISPR sequence as a reference for equivalent orientation. The NGC and GAA motifs are disclosed for subtype I-D and I-B respectively.
(TIF)

Figure S3 **Alignment of the CRISPR sequence of eHP-16 fosmid and the most similar CRISPRs found in the nr/nt collection of GenBank database.** When CRISPRs are located in a chromosome, only the name of the harboring strain is indicated and when in a plasmid, the name of the replicon is also shown between brackets. Mismatches with respect to the CRISPR in the fosmid are labeled in red.
(TIF)

Figure S4 **Genomic organization of the non classified fosmids.** Colour code as in Figure 1.
(TIF)

Table S1 **Predicted tRNA and ORFs of the viral fosmids.**
(XLSX)
