# Supplementary figures and images for: Reconstructing Viral Genomes from the Environment Using Fosmid Clones: The Case of Haloviruses
Source: PLoS One. 2012 Mar 30;7(3):e33802. doi: 10.1371/journal.pone.0033802 (PMC3316494; doi:10.1371/journal.pone.0033802)

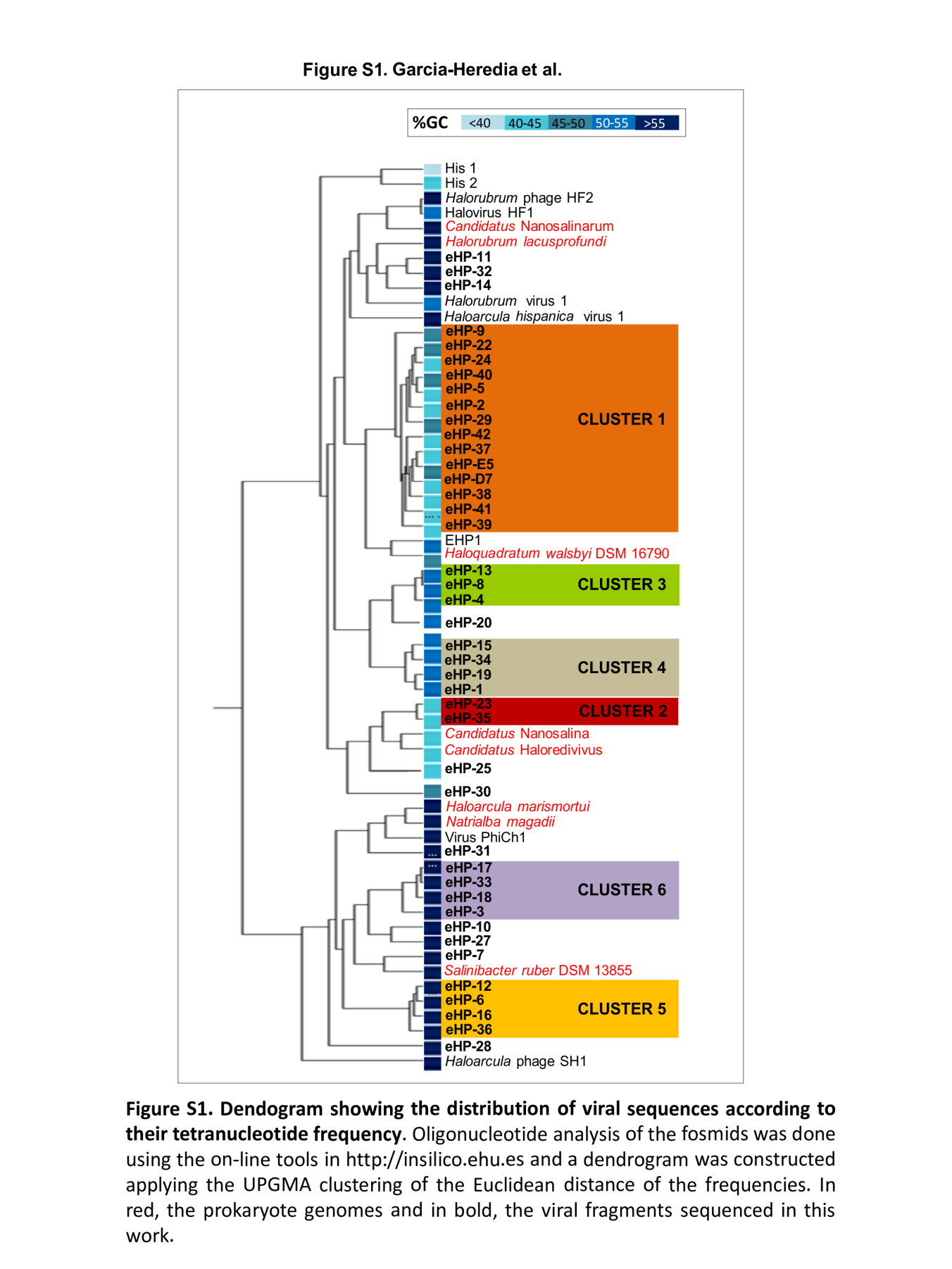

Supplement: Figure S1 — Dendogram showing the distribution of viral sequences according to their tetranucleotide frequency. Oligonucleotide analysis of the fosmids was done using the on-line tools in http://insilico.ehu.es and a dendrogram was constructed applying the UPGMA clustering of the Euclidean distance of the frequencies. In red, the prokaryote genomes and in bold, the viral fragments sequenced in this work. (TIF) [file pone.0033802.s001.tif]

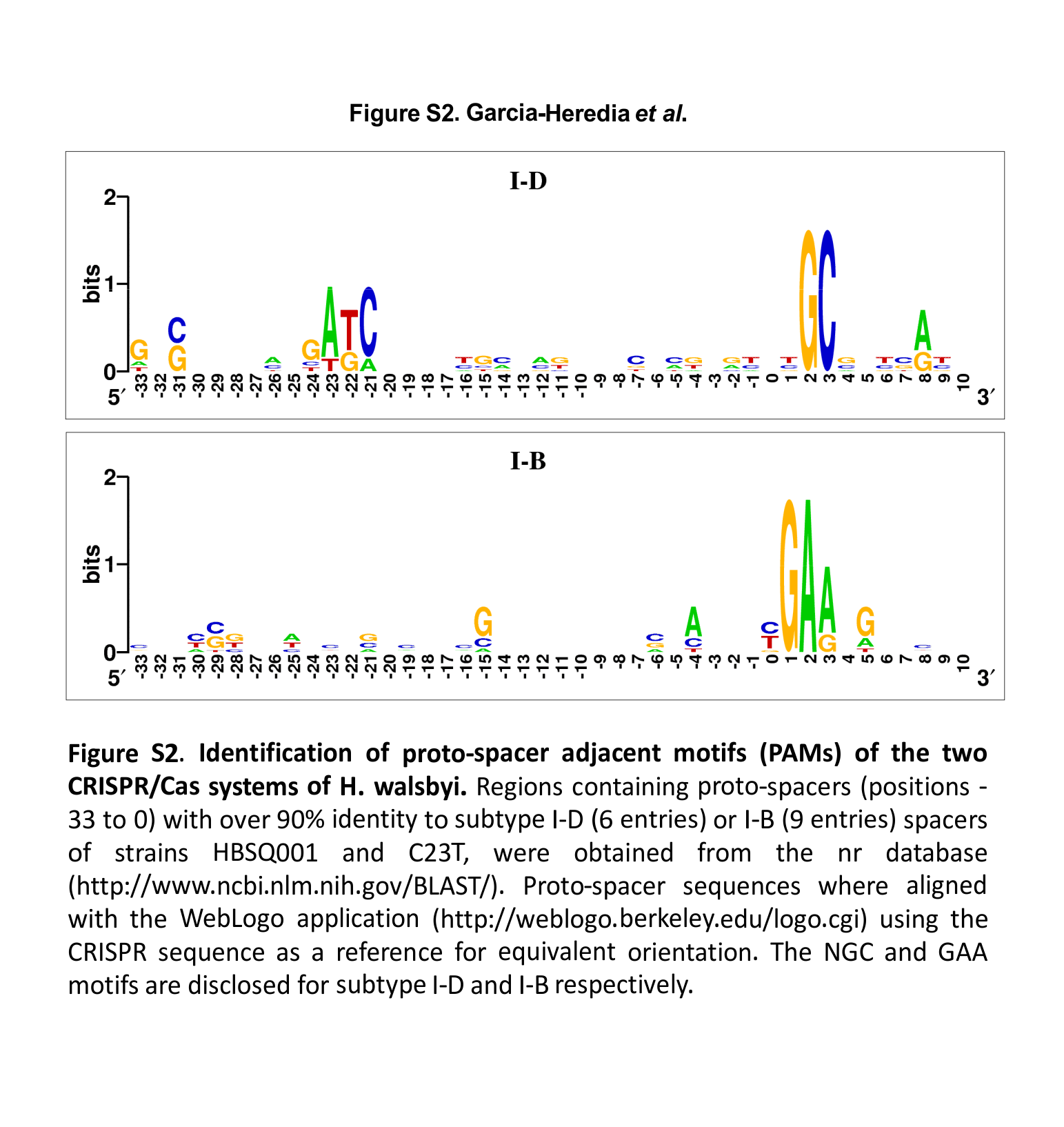

Supplement: Figure S2 — Identification of proto-spacer adjacent motifs (PAMs) of the two CRISPR/Cas systems of H. walsbyi . Regions containing proto-spacers (positions −33 to 0) with over 90% identity to subtype I-D (6 entries) or I-B (9 entries) spacers of strains HBSQ001 and C23T, were obtained from the nr database (http://www.ncbi.nlm.nih.gov/BLAST/). Proto-spacer sequences where aligned with the WebLogo application (http://weblogo.berkeley.edu/logo.cgi) using the CRISPR sequence as a reference for equivalent orientation. The NGC and GAA motifs are disclosed for subtype I-D and I-B respectively. (TIF) [file pone.0033802.s002.tif]

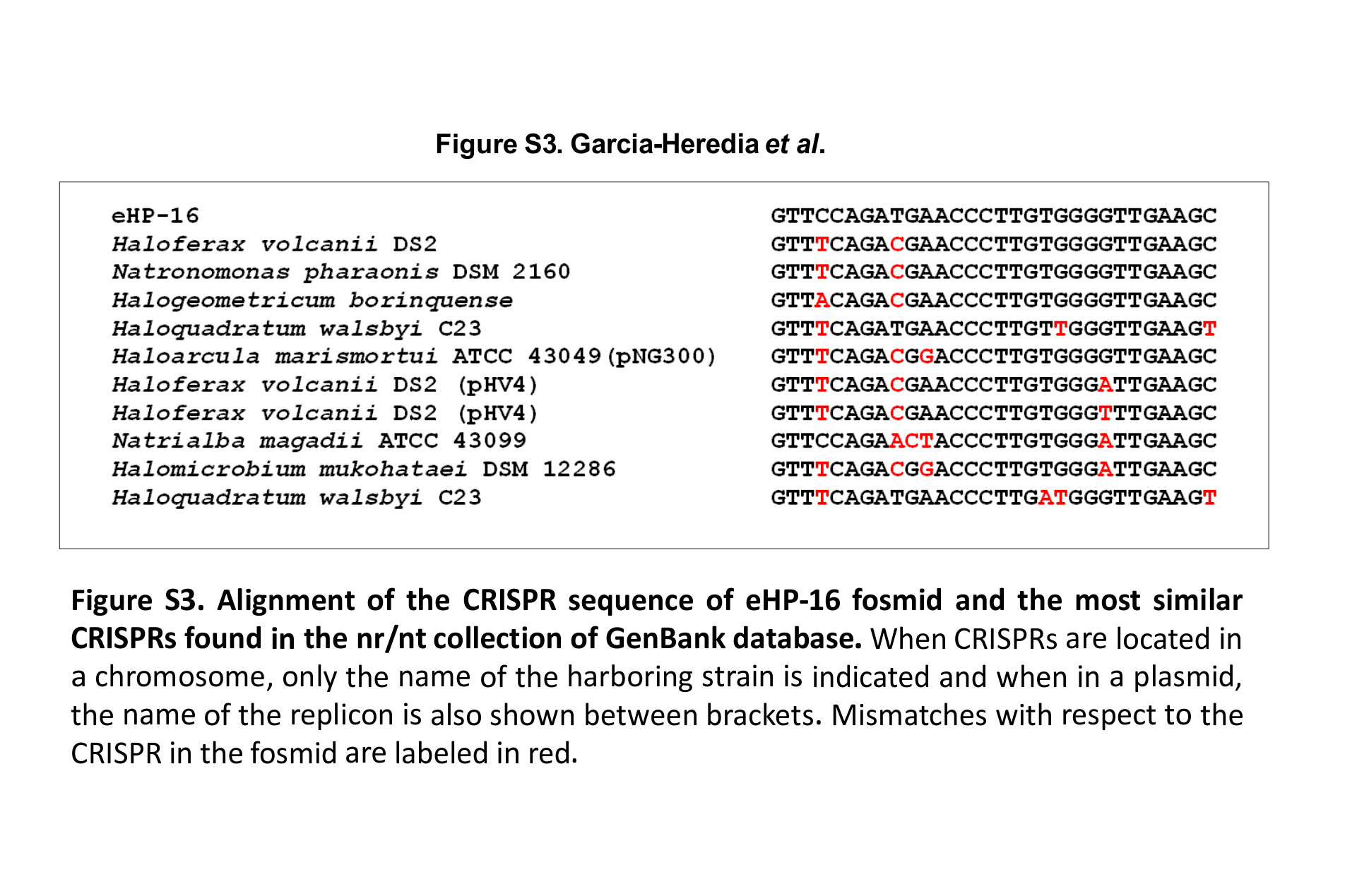

Supplement: Figure S3 — Alignment of the CRISPR sequence of eHP-16 fosmid and the most similar CRISPRs found in the nr/nt collection of GenBank database. When CRISPRs are located in a chromosome, only the name of the harboring strain is indicated and when in a plasmid, the name of the replicon is also shown between brackets. Mismatches with respect to the CRISPR in the fosmid are labeled in red. (TIF) [file pone.0033802.s003.tif]

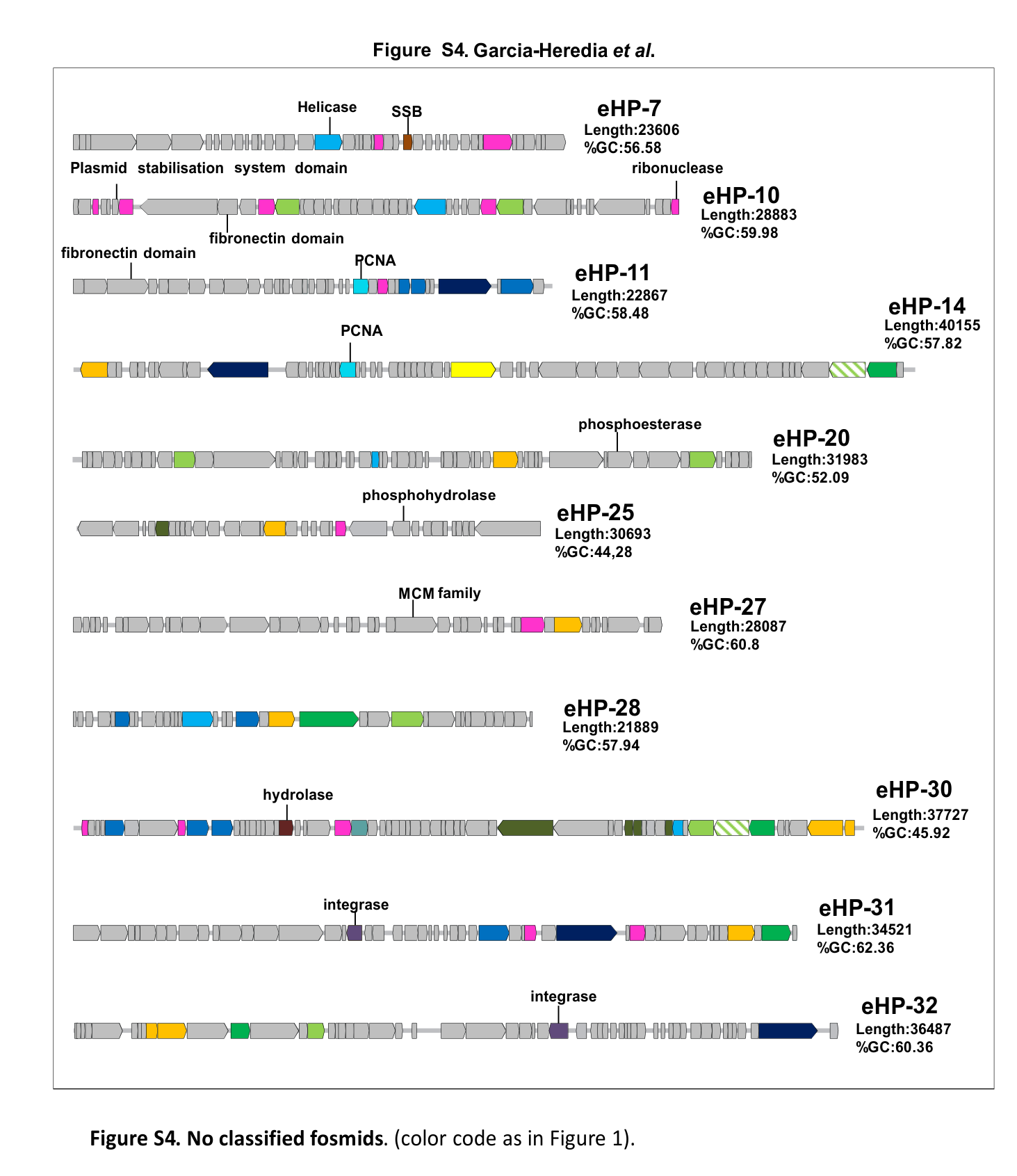

Supplement: Figure S4 — Genomic organization of the non classified fosmids. Colour code as in Figure 1. (TIF) [file pone.0033802.s004.tif]
